# Effect of Whey-Derived Lactopeptide β-Lactolin on Memory in Healthy Adults: An Integrated Analysis of Data from Randomized Controlled Trials

**DOI:** 10.1007/s12603-022-1733-8

**Published:** 2022-01-28

**Authors:** Takafumi Fukuda, A. Kanatome, A. Takashima, O. Tajima, S. Umeda, Y. Ano

**Affiliations:** 1 KIRIN Central Research Institute, Kirin Holdings Company Limited, 26-1, Muraoka-Higashi 2-chome, 251-8555, Fujisawa, Kanagawa, Japan; 2 Faculty of Science, Gakushuin University, 171-8588, Tokyo, Japan; 3 Department of Psychology, Keio University, Mita, Minato-ku, 108-8345, Tokyo, Japan

**Keywords:** β-lactolin, cued recall, dairy products, dopamine, frontal lobe

## Abstract

**Context:**

Epidemiological studies have shown that consumption of dairy products reduces the risk of dementia and cognitive decline in older individuals. Tryptophan-tyrosine-related β-lactopeptides and their representative β-lactolin of glycine-threonine-tryptophan-tyrosine tetra-peptide have been identified as agents in dairy products, which improve cognitive function as well as memory function via the activation of the dopaminergic system in a mouse model of amnesia. Previous clinical trials have shown that supplementation with β-lactolin improves memory retrieval in healthy older adults. Specifically, β-lactolin improved the scores in some neuropsychological tests. However, the effects of β-lactolin on memory function have not been clarified.

**Objectives:**

The aim of this study was to evaluate the effect of β-lactolin on memory function using statistical methods.

**Data Sources:**

We searched the Web of Science, Cochrane Library, and JDream III until November 2021 to identify relevant randomized controlled trials for integrated analysis.

**Data Synthesis:**

Three randomized controlled trials evaluating the effect of β-lactolin on memory in healthy adults were selected for the integrated analysis. The results showed that the score of cued recall among the neuropsychological tests in the β-lactolin group was significantly higher than that in the placebo group (g=0.33; 95% CI: 0.10, 0.55). In addition, the total memory score was higher but this difference was not significant (g=0.17; 95% CI: −0.09, 0.43).

**Conclusions:**

Taken together, these results suggest that supplementation with β-lactolin improves cued recall in healthy older adults.

## Introduction

**W**ith the increasing age in society, the number of people with age-related cognitive impairment and dementia is rapidly increasing (1). Due to the lack of a fundamental therapeutic approaches, prevention of cognitive decline and dementia in daily life has received increasing attention.

Memory decline and memory impairment are the main symptoms in patients with dementia or mild cognitive impairment. Memory function consists of three processes: memory acquisition, memory retention, and memory recall (2). Memory recall is categorized into free recall, cued recall, and recognition (2). Since the degree of cognitive decline and the stage of dementia are related to each recall ability, evaluating each recall ability separately to understand the preventive and therapeutic effects provides useful information (Bastin and Salmon, Grober et al., 2000, Ivanoiu, 2005, Salmon et al., 2008). For instance, Bastin and Salmon reviewed that cued recall ability decline appears in the early stage of dementia (3). Furthermore, it has been reported that frontal lobe function is greatly affected by aging, and changes in this region appear earlier than in other brain regions (Salat et al., 1999, Raz, 1997). The frontal lobe is responsible for memory retrieval and working memory (9). Clinical studies in older adults have suggested that activating the frontal lobe function can delay cognitive decline with age (Shatil et al., 2014, Sandrini, 2016). Specifically, these reports suggested that maintenance of frontal lobe function in daily life was effective in preventing cognitive decline and dementia.

In recent years, evidence has been accumulated for the prevention of cognitive decline by dietary habits. For instance, Wu and Sun showed the efficacy of Mediterranean diet, which is characterized by a high consumption of fruits, vegetables, and fish and low consumption of meat and saturated fatty acids, in reducing the risk of cognitive disorders by meta-analysis (12). Further, the dietary habits have been shown to be associated with the biomarker of Alzheimer's disease (13). In addition, some studies indicated that specific dietary components such as long-chain omega-3 fatty acids (Yanai, 2017, Dyall, 2015) and curcumin (Goozee, 2016, Reddy, 2018) have the potential for prevention of cognitive decline. Recent epidemiological studies in Japan have shown that dietary pattern is associated with the risk of dementia (18), and intake of dairy products in daily life particularly prevents cognitive decline and reduces the risk of dementia in old age people (19). Based on these studies, we have previously identified the β-lactopeptide tryptophan-tyrosine (WY)-related lactopeptides, including WY and glycine-threonine-tryptophan-tyrosine (GTWY) (20). In particular, β-lactolin is a GTWY tetrapeptide derived from β-lactoglobulin, which is abundant in dairy products fermented with Penicillium fungi, such as camembert cheese. Orally administered β-lactolin is delivered to the brain and inhibits the activity of monoamine oxidase B (MAO-B) in mice, thus increasing dopamine levels in the frontal cortex and hippocampus (Ano, 2018, Ayabe et al., 2019). It was shown that β-lactolin improves spatial working (20), episodic object recognition memory (21), and prefrontal cortex (PFC)-associated reversal discrimination learning in mice (22). In addition, β-lactolin prevents memory impairment and suppresses neural inflammation in aged mice (23) and Alzheimer's transgenic 5×FAD mice (24).

In addition to preclinical studies, our previous randomized controlled trials (RCTs) showed that supplementation with β-lactolin improved the score of the verbal fluency test (VFT), which evaluated memory retrieval in healthy middle-aged adults compared with the placebo group (Kanatome, 2021, Kita et al., 2018). Furthermore, in one of these RCTs, the enhancement of P300 event-related potential amplitude, which reflects neural activity, was observed in the β-lactolin group compared with the placebo group (25). In another RCT that evaluated memory retrieval in healthy older adults, supplementation with β-lactolin improved visual paired-associate learning (27). In addition, two RCTs have shown that frontal lobe cerebral blood flow during cognitive tasks was increased in the β-lactolin group compared to the placebo group (Ano et al., 2020, Ano et al., 2021). These trials suggest that supplementation with β-lactolin enhances neural activity and increases cerebral blood flow, resulting in improved memory retrieval, especially cued recall. However, statistical evidence regarding the effects of β-lactolin on memory retrieval is lacking. Previous clinical trials have evaluated various cognitive functions and showed that supplementation with β-lactolin improved neuropsychological tests. In the current study, we statistically evaluated the efficacy of β-lactolin on global memory function, cued recall, and free recall using previous RCT data on statistical methods used in meta-analysis.

## Methods

### Search Strategy

We searched the literature reported between 1950 and November 1, 2021 in the Web of Science, Cochrane Library, and JDream III (JSTPlus+JMEDPlus+JST7580). The search terms are listed in Tables S1 and S2. JDream III were searched in Japanese.

### Study selection

The inclusion criteria were as follows: 1) participants-healthy adults; 2) study design-RCTs; 3) type of intervention included studies comparing the effect of β-lactolin with the placebo on cognitive function; and 4) outcomes were results of memory performance tests. The exclusion criteria were as follows: 1) reports not written in English or Japanese; 2) non-eligible publication types such as review articles, conference abstracts, and books; or 3) duplicate reports of the same study.

Primary screening (title and abstract) and secondary screening (full text) were independently performed by two reviewers (A.K. and T.F.).

### Outcome measures

The primary outcome of this systematic review was memory ability. Cued recall ability was evaluated by associate learning, and so forth, as the secondary outcome based on the mechanism by which β-lactolin stimulates the dopaminergic system. The neuropsychological tests that assessed cued recall ability were classified by independent cognition experts. Whenever clinical trials referred to the same population at different follow-up periods, we used only the population with the longest follow-up period to avoid data duplication.

### Statistical analysis and data synthesis

We used Review Manager ver.5.3.5 (The Nordic Cochrane Center, The Cochrane Collaboration) to conduct the analysis. The standardized mean differences (SMD, calculated as Hedges' g adjusted for small sample bias) of the mean change from baseline were used (30). When several outcomes were obtained in the same study, combined measurements were calculated to provide a single quantitative measurement for our integrated analysis (30). For example, if a study reported two relevant outcomes, expressed as two effect sizes (g_1_ and g_2_), the overall mean effect size for the composite measure was as follows:

$$\bar{g}=\frac{1}{2}(g_{1}+g_{2}).$$

The variance (V) of the composite effect size was calculated as follows:

$$V_{\bar{g}}={\left(\frac{1}{2}\right)}^{2}\left(V_{g_{1}}+V_{g_{2}}+2r\sqrt{V_{g_{1}}}\sqrt{V_{g_{2}}}\right)$$

where r is the correlation coefficient between the two outcomes. Individual SMD values were combined using the inverse variance random-effects method. The pooled effect sizes and 95%CI are shown in a forest plot. Heterogeneity was assessed using I^2^: 1) very low- 0 to < 25%, 2) low- 25 to < 50%, 3) moderate-50 to < 75%, and 4) large-75 to 100%; and the Cochrane's Q statistic, which is considered significant for P < 0.05.

## Results

### Description of studies

**Figure 1:**
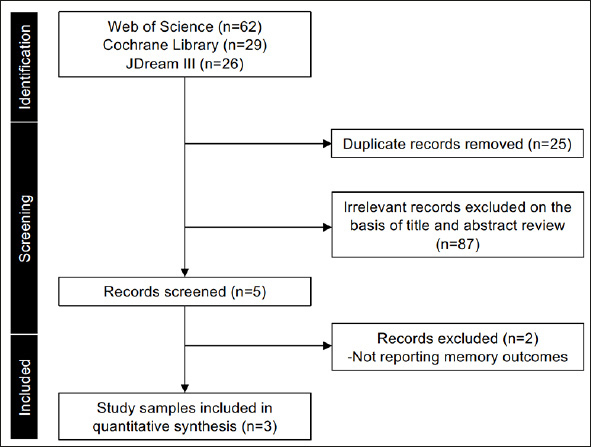
Flow diagram of the study selection process

Following the initial literature review, 117 studies were found, as shown in Figure 1. Of these, 114 were excluded, and 3 studies met the eligibility criteria (Kanatome, 2021, Kita et al., 2018, Kita, 2019). The characteristics of the studies included in the integrated analysis are presented in Table 1. All studies reported memory ability and cued recall ability assessment outcomes (Table 2). Three studies were reported by the same group. One of the three studies was conducted in Osaka, and the other two were conducted in Tokyo, Japan. Upon contacting the authors of each paper, it was found that the possibility of duplication of participants was extremely low because the screening sources of the participants in each study were different.

**Table 1:** Characteristics of the included studies

**Author (year)**	**RCT Design**	**Setting**	**Blinding States**	**No. of Participants ITT; Completed (Male/ Female)**		**Participants' Condition**	**Participants' Age Mean (SD)**		**Test Foods Form**		**Intake Duration (weeks)**	**Interval Assessed (weeks)**
				**β-lactolin**	**Placebo**		**β-lactolin**	**Placebo**	**β-lactolin**	**Placebo**		
Kita (2018)	Parallel	Osaka, Japan	Double-blinded	50; 48 (17/31)	51; 50 (17/33)	Healthy	52.3 (4.3)	51.8 (5.2)	Tablet (β-lactolin: 1.6 mg)	Tablet (Maltodextrin)	12	0, 6, 12
Kita (2019)	Parallel	Tokyo, Japan	Double-blinded	57; 51 (17/34)	57; 53 (20/33)	Healthy	60.7 (5.7)	61.2 (5.6)	Tablet (β-lactolin: 1.6 mg)	Tablet (Maltodextrin)	12	0, 6, 12
Kanatome (2021)	Parallel	Tokyo, Japan	Double-blinded	15; 15 (6/9)	15; 15 (7/8)	Healthy	53.3 (5.2)	53.0 (5.2)	Tablet (β-lactolin: 1.6 mg)	Tablet (Dextrin)	6	0, 6


ITT, Intent to treat; RCT, Randomized controlled trial; SD, standard deviation

**Table 2:** List of memory and learning tests and change scores from baseline to final evaluation point

**Study**		**Memory and learning test**	**Category**	**Change scores from baseline (SD)**	
				**β-lactolin group**	**Placebo group**
Kita, 2018	Word recall	Immediate	Free recall	0.8 (1.3)	0.9 (1.1)
		5 min delayed	Free recall	1.4 (1.7)	1.7 (1.3)
		20 min delayed	Free recall	1.2 (1.7)	1.3 (1.6)
	Story recall	Immediate	Free recall	1.41 (3.38)	1.63 (3.63)
		20 min delayed	Free recall	2.40 (3.66)	2.24 (3.65)
	Verbal fluency	Beginning with «a»	Cued recall	2.8 (3.1)	1.7 (3.1)
		Animal names	Cued recall	1.9 (3.9)	1.4 (3.9)
Kita, 2019	WMS-R	Design memory	Recognition	0.24 (1.73)	0.28 (1.38)
		Logical memory I	Free recall	2.3 (5.1)	1.5 (4.8)
		Visual paired associates I	Cued recall	1.9 (3.1)	0.7 (2.1)
		Logical memory II	Free recall	3.0 (5.3)	2.5 (5.2)
		Visual paired associates II	Cued recall	0.10 (1.00)	0.02 (0.80)
	S-PA	1st	Cued recall	0.57 (2.43)	0.30 (2.23)
		2nd	Cued recall	1.08 (2.93)	0.11 (1.94)
		3rd	Cued recall	0.55 (2.63)	0.17 (2.35)
	RBANS	Immediate memory	Free recall	1.48 (9.52)	1.36 (8.44)
		Language	Free recall	3.94 (10.33)	3.08 (8.74)
		Delayed memory	Free recall	3.62 (11.35)	4.21 (11.51)
		RMT-F	Recognition	0.058 (0.119)	0.078 (0.138)
Kanatome, 2021	Verbal fluency	Beginning with «a»	Cued recall	1.9 (2.2)	0.0 (2.4)
		Beginning with «shi»	Cued recall	1.9 (3.6)	0.1 (2.7)
		Animal names	Cued recall	1.5 (3.4)	−0.2 (1.7)


RBANS, Repeatable Battery for the Assessment of Neuropsychological Status; RMT-F, Recognition memory test for faces; SD, standard deviation; S-PA, Standard verbal paired-associate learning test; WMS-R, Wecheler Memory Scale-Revised

### Participant characteristics

The total number of participants included was 232 (β-lactolin: n=114, mean group size: n=38.0, placebo: n=118, mean group size: n=39.3), and the summary characteristics of each study are presented in Table 1. The drop-out rates were 6.6% in the β-lactolin group and 4.1% in the placebo group. The average age of participants in both condition was 56.2 years and 36.2% of all participants were male.

### Interventions

In all studies, β-lactolin and the placebo containing dextrin or maltodextrin instead of β-lactolin were provided to participants as tablets, and the dose of β-lactolin was 1.6 mg per day for 6 or 12 weeks (Table 1).

### Outcome measures

Outcome measures were summarized in Table 2 and Table S3 for details. Kita et al. (2018) used seven tests to assess memory. The verbal fluency test (31), in which the ability of retrieval from long-term memory according to phonemic or semantic cues, was performed to evaluate cued recall ability. The assessments of memory function were conducted at baseline, week 6, and week 12. The changes from baseline to week 12 were analyzed in our study. All of tests were conducted in Japanese by well-trained clinical staff who were not dependent on the sponsor. Kita et al. (2019) conducted 12 memory tests to assess the cued recall ability using visual paired associates I and II from the Wechsler Memory Scale-Revised (32). Subjects were presented pictures with associated colors, and were then required to point to the correct colors when presented with each of the pictures, immediately (Visual paired-associate I) and 30 min later (Visual paired-associate II). They also used the Standard verbal paired-associate learning test (33), in which subjects were presented with pairs of words that were semantically unrelated. S-PA were conducted at baseline, week 6, and week 12. The other tests were assessed at baseline and week 6. The changes from baseline to week 12 were analyzed in our study. All of tests were conducted in Japanese by well-trained clinical staff who were not dependent on the sponsor. In addition, Kanatome et al. (2021) performed three types of verbal fluency to evaluate cued recall. The evaluations were conducted at baseline and week 6, and the changes from baseline to week 6 were analyzed in our study. All of tests were conducted in Japanese by well-trained clinical staff who were not dependent on the sponsor.

### Memory function (all)

Multiple outcome measures for memory function were reported in each trial. Therefore, composite effect size (Hedges' g statistics) and variance were computed according to the above equations (Table 3).

**Table 3:** Computed composite score of each study

**Study**	**Memory Type**	**Hedges' g**	**Variance (V)**	**P-value**
Kita, 2018	All	0.02	0.010	0.87
	Cued recall	0.23	0.022	0.13
	Free recall	−0.07	0.016	0.60
Kita, 2019	All	0.06	0.006	0.41
	Cued recall	0.24	0.015	0.054
	Free recall	−0.05	0.014	0.67
Kanatome, 2021	All	0.65	0.044	0.002
	Cued recall	0.65	0.044	0.002

**Figure 2:**
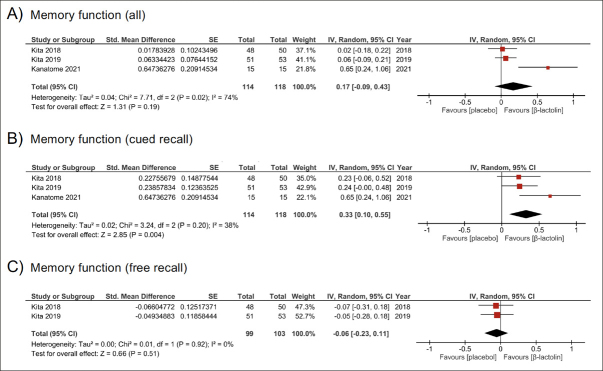
Efficacy of β-lactolin on memory function (A) Efficacy of β-lactolin on measurements of memory parameters, (B) Efficacy of β-lactolin on measurements of cued recall, (C) Efficacy of β-lactolin on measurements of free recall; Effect estimates are based on a random-effects model, and studies are rank-ordered by year of publication.

As a result of integrating the effect sizes by the meta-analysis method, the effect size of β-lactolin compared with the placebo was positive, but not significant. (g=0.17; 95% CI −0.09 to 0.43, z=1.31, P=0.19) with moderate heterogeneity (I^2^=74%, P=0.02) (Figure 2A).

### Memory function (cued recall)

The composite effect size for cued recall was computed to evaluate the efficacy of β-lactolin on cued recall (Table 3). The integrated analysis revealed that β-lactolin significantly improved cued recall compared with the placebo (g=0.33; 95% CI 0.10 to 0.55, z=2.85, P=0.004), with low heterogeneity between studies (I2=38%, P=0.20) (Figure 2B).

### Memory function (free recall)

The composite effect size for free recall was computed to evaluate the efficacy of β-lactolin on free recall (Table 3). β-lactolin had no significant effect on free recall compared to the placebo (g=−0.06; 95% CI −0.23 to 0.11, z=0.66, P=0.51), with low heterogeneity between studies (I^2^=0%, P=0.92) (Figure 2C).

## Discussion

This study was conducted to investigate the effects of β-lactolin on memory retrieval, especially cued recall. The integrated analysis revealed that supplementation with β-lactolin for 6–12 weeks significantly improved cued recall rather than free recall, and recognition in healthy middle-aged to older adults.

In this review, we categorized each neuropsychological test for memory function used to produce outcomes in the clinical trials into free recall, cued recall, and recognition according to each assessment method. The studies included verbal fluency test (semantic/category fluency ‘words categorized to animal names' and phonemic/letter fluency ‘words beginning with a' or shi'), visual paired associates I and II (Wechsler Memory Scale-Revised) and standard verbal paired-associate learning test to assess cued recall ability. As a result of the integrated analysis, the scores of cued recall were significantly higher in the β-lactolin group than in the placebo group.

A preclinical study indicated that β-lactolin penetrated the brain and inhibited MAO-B, resulting in increased dopamine and activation of the dopaminergic system in the cortex and improved PFC-associated reversal discrimination learning in the touch screen operant test (22), and spatial memory test (20). In a clinical trial, supplementation with β-lactolin increased regional cerebral blood flow in the left dorsal PFC area (Ano et al., 2020, Ano et al., 2021). These studies indicate that β-lactolin activates the function of the frontal cortex area and improves cognitive function associated with frontal lobe function. There is some evidence indicating that the dopaminergic system in the frontal lobe is involved in cued recall ability. A study using dopamine transporter heterozygous knockout mice (DAT [+/−]) with chronically insufficient dopamine reuptake activity indicated that dopamine imbalance caused deficits in associative spatial memory recall (34). It was also shown that dopamine is necessary for cue-dependent memory retrieval in fear-conditioning tests using dopamine deficient mice (35). Recent studies have shown that dopamine D1-like receptors in the PFC regulate contextual cued recall. Treatment with SCH23390, an antagonist of the dopamine D1-like receptor, in the dorsomedial PFC attenuates contextual memory retrieval (36). Our previous study also indicated that inoculation with SCH23390 attenuated the effect of β-lactolin on memory (20). Furthermore, Clos et al. revealed that dopamine enhanced associative recall rather than the encoding process in a clinical study in healthy adults using the dopamine D2-like receptor antagonist haloperidol (37). Interestingly, left lateral PFC activity was linked with higher performance of association memory (37). This result is consistent with that of our study of cerebral blood flow. These reports indicate that dopamine in the PFC is crucially associated with cued recall. Taken together, these results suggest that β-lactolin improves memory retrieval, especially cued recall, through activation of the dopaminergic system.

Frontal lobe function is impaired in Alzheimer's disease (Chen, 2001, Perry and Hodges, 1999) and other types of dementia (40). Some studies have shown that frontal lobe dysfunction is observed in MCI (Saykin, 2004, Liang et al., 2011). Furthermore, it has been suggested that cued recall performance, including verbal fluency, is associated with cognitive function decline in dementia patients (Rentz, 2013, Cottingham and Hawkins, 2010). A preclinical study using mouse models demonstrated that β-lactolin supplementation prevented age-related cognitive decline (23) and progression of Alzheimer's disease (24). In particular, the cognitive functions related to the frontal lobe were maintained. Investigating the preventive effect of β-lactolin, which activates the frontal lobe in humans with dementia, is recommended in future studies.

This study has several limitations. First, the selected clinical studies cited hereinwere reported by the same research group. Second, only three studies were included. Future studies are required to substantiate our conclusions. In particular, although there was no significant difference in the effect of β-lactolin on overall memory function, it showed higher scores than the placebo. Therefore, it is necessary to confirm the effectiveness of β-lactolin on overall memory function after ensuring an appropriate sample size in future studies. Finally, we evaluated memory function to investigate the effect of β-lactolin on frontal lobe function. Further statistical analysis should be conducted on other neuropsychological tests related to frontal lobe function, such as attention and executive function tests.

In conclusion, the intake of β-lactolin from whey-derived lactopeptide in daily life is an easy and safe approach. It would be welcome as a novel approach to support the frontal cortex associated function, especially cued memory recall, which might contribute to preventing cognitive decline and dementia in an aging society.

## Electronic supplementary material

Supplementary material, approximately 16 KB.
